# DNA methylation patterns in tissues from mid-gestation bovine foetuses produced by somatic cell nuclear transfer show subtle abnormalities in nuclear reprogramming

**DOI:** 10.1186/1471-213X-10-27

**Published:** 2010-03-07

**Authors:** Christine Couldrey, Rita SF Lee

**Affiliations:** 1 AgResearch, Reproductive Technologies Group, Ruakura Research Centre, East Street, Private Bag 3123, Hamilton, New Zealand

## Abstract

**Background:**

Cloning of cattle by somatic cell nuclear transfer (SCNT) is associated with a high incidence of pregnancy failure characterized by abnormal placental and foetal development. These abnormalities are thought to be due, in part, to incomplete re-setting of the epigenetic state of DNA in the donor somatic cell nucleus to a state that is capable of driving embryonic and foetal development to completion. Here, we tested the hypothesis that DNA methylation patterns were not appropriately established during nuclear reprogramming following SCNT. A panel of imprinted, non-imprinted genes and satellite repeat sequences was examined in tissues collected from viable and failing mid-gestation SCNT foetuses and compared with similar tissues from gestation-matched normal foetuses generated by artificial insemination (AI).

**Results:**

Most of the genomic regions examined in tissues from viable and failing SCNT foetuses had DNA methylation patterns similar to those in comparable tissues from AI controls. However, statistically significant differences were found between SCNT and AI at specific CpG sites in some regions of the genome, particularly those associated with SNRPN and KCNQ1OT1, which tended to be hypomethylated in SCNT tissues. There was a high degree of variation between individuals in methylation levels at almost every CpG site in these two regions, even in AI controls. In other genomic regions, methylation levels at specific CpG sites were tightly controlled with little variation between individuals. Only one site (HAND1) showed a tissue-specific pattern of DNA methylation. Overall, DNA methylation patterns in tissues of failing foetuses were similar to apparently viable SCNT foetuses, although there were individuals showing extreme deviant patterns.

**Conclusion:**

These results show that SCNT foetuses that had developed to mid-gestation had largely undergone nuclear reprogramming and that the epigenetic signature at this stage was not a good predictor of whether the foetus would develop to term or not.

## Background

Somatic cell nuclear transfer (SCNT) has been used to successfully produce cloned animals from several mammalian species since a sheep was cloned using a differentiated somatic donor cell [1]. However, to date widespread application of SCNT in agricultural breeding programs has not yet been captured because the technology remains inefficient despite more than 10 years of research. Irrespective of the species being cloned, there is still a high rate of pregnancy failure throughout gestation [2-6]. The most common SCNT foetal phenotypes across species are foetal overgrowth and loss of allometric growth regulation (collectively known as "large offspring syndrome"), musculoskeletal defects, and acute, excessive accumulation of allantoic fluid (hydrallantois or hydrops) accompanied by perturbations in the composition of this fluid [7]. In cattle, the large offspring syndrome appears to be independent of the donor cell genetics.

Many of the developmental defects observed in cloned bovine foetuses suggest the involvement of growth regulating genes, particularly those known to be imprinted. Some of these genes play key roles in regulating cellular proliferation, growth and development of the foetus and the placenta (reviewed [8]). The phenotypes commonly observed in SCNT foetuses bear many similarities to some of those seen in experimentally-created imprinting disruptions in mice (silencing of both alleles or biallelic expression of imprinted genes), or to naturally-occurring human syndromes, such as Beckwith-Wiedemann syndrome (BWS) [9-13]. These similarities suggest that the expression of some of these imprinted genes is abnormal and/or that these genes are not appropriately reprogrammed following SCNT. Furthermore, SCNT calves dying shortly after birth were shown to have abnormal expression of imprinted genes in a variety of organs when compared to controls generated by AI [14]; this was not the case in surviving adult clones [15,16]. During the development of multicellular organisms, different cells and tissues acquire different programs of gene expression. It is thought that a substantial part of this gene regulation is mediated through epigenetic modifications such as DNA methylation, histone tail modifications and the binding of non-histone proteins to chromatin [17-19] so that each somatic cell in the organism has its own epigenetic signature (epigenome) which reflects its genotype, developmental history and environmental influences, which ultimately determines the phenotype of the cell and the organism. This is clearly illustrated in the events following fertilization, where the majority of the genome undergoes active paternal demethylation, then passive maternal demethylation. Re-methylation of the genome then occurs during repeated mitosis as cells progress towards lineage commitment and the development of the embryo proper and the placenta [18,20-22]. How the developmental programs are coordinated and orchestrated from the genomic blueprint is still poorly understood, even in normal development.

In reproductive cloning by SCNT, the epigenetic signature of a differentiated somatic cell must be reset to a state resembling totipotency, capable of driving full development after fusion of the cell with an enucleated oocyte cytoplast. Incomplete nuclear reprogramming is widely postulated to be a major contributor to the low developmental success rate following SCNT. Evidence to support this include observed hypo-methylation [23-26], hyper-methylation [27,28], or mosaic methylation states [29] in tissue samples collected from abnormal foetuses or cloned calves that died shortly after birth. Normal methylation following SCNT has also been reported [28,30,31], suggesting a degree of stochasticity in nuclear reprogramming. The variable findings from different studies are due to different genes or genomic regions examined, different tissues used and controls that were not gestation- or age-matched. This has made comparisons between previous studies difficult.

Cloned animals that reach maturity are able to reproduce normally and give rise to normal offspring without the high rate of pregnancy failure or large offspring syndrome associated with SCNT [32,33], suggesting that underlying cause/s of the abnormalities associated with SCNT is/are epigenetic.

In this study, we examined the DNA methylation patterns in a panel of candidate genes using tissues from three foetal organs (liver, kidney and adrenal glands) of similar gestations generated by either SCNT or AI. These organs were selected because of the pivotal roles they play in foetal metabolism, nutrient sensing and hematopoiesis (liver), regulation of blood pressure and foetal fluid homeostasis (kidney) and foetal endocrinology (adrenal glands). As most cases of hydrallantois occur from or just after mid-gestation, the selection of this stage of gestation allowed us to compare samples from foetuses that showed clear physiological and anatomical abnormalities with those that had not yet exhibited these symptoms and thus, had the potential to develop to further.

The genes selected consisted of those known to be imprinted in other species, non-imprinted genes and repeat sequences (satellites 1, 2 and alpha). The imprinted genes include those associated with BWS, such as IGF2, KCNQ1, CDKN1C, KCNQ1OT1; others such as ASCL2, HAND1, DIO3; and SNRPN, a gene in the Prader-Willi and Angelman syndrome locus. The non-imprinted genes include colony stimulating factor (CSF-1), STAT5a, DKK-1, and GR, which codes for the glucocorticoid receptor, that mediates the action of glucocorticoids and mineralcorticoids, both important in regulation of fluid composition. The CpG islands examined included those located upstream of transcriptional start sites or within the gene itself or in equivalent regions shown in other species to be differentially methylated regions (DMRs) and normally associated with imprinted genes. The SNRPN site is equivalent to the imprinting centre (IC) of the human gene [34] which has been shown to be aberrantly methylated in the Prader-Willi (PWS) and Angelman syndromes [35]. The KCNQ1OT1 region is equivalent to the human KCNQ1OT1 DMR which was found to be hypomethylated at increased frequency in human IVF offspring [12,36,37]. Together, this selection of genomic sequences allowed us to assess how well these different regions were re-programmed after nuclear transfer in embryos that were capable of developing at least to mid-gestation.

## Results

### Pregnancy rates in SCNT and AI

From the first ultrasound scan at Day 35, 29 of the 42 (69%) SCNT recipient dams that received a Day 7 blastocyst were found to be pregnant. By Day 130, just 10 days prior to the development of the first SCNT hydrops case, only 14 were still pregnant (33%). Four subsequently developed clinical hydrops and were slaughtered. For the AI group, 13 of the 18 (72%) recipients inseminated were pregnant at Day 35, 11 of these still pregnant (61%) at Day 130; none showed signs of abnormal fluid accumulation.

### DNA methylation analysis

DNA methylation analysis at each of the listed genomic regions was performed from the same genomic DNA for each sample. Because the cleavage of the transcribed RNA is sequence-specific, some fragments may contain only one CpG site whilst others may contain up to 6 CpG sites where the CpG dinucleotides were arranged consecutively in the sequence or close together. Where multiple CpG sites occurred within a fragment, the methylation level reported by the EpiTYPER software was that of the most highly methylated site. Where is was not possible to resolve two fragments with the same mass but with different sequences, the methylation level is recorded as an average of the two fragments. The number of CpG sites or group of sites that could be analyzed for each region is given in each figure legend.

### Imprinted genes

#### IGF2 exon 10

**Figure 1:**
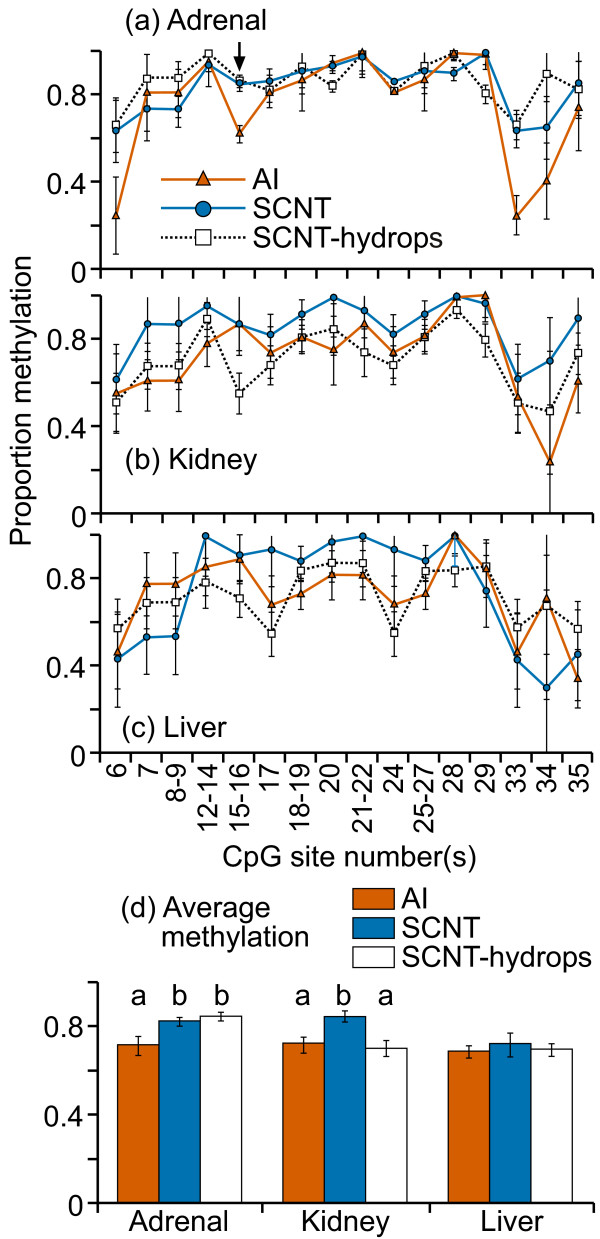
**DNA methylation at the IGF2 exon 10 DMR**. Sixteen cleavage fragments containing 24 out of a total of 34 CpG sites in this region could be analyzed. The pairs of cleavage fragments containing CpG sites 6 and 23; 7 and 8-9; 17 and 24; 18-19 and 25-27; could not be distinguished from each other in the analysis. Therefore, the proportion of methylation in each of these fragment pairs is represented as an average value.

The CpG island located in IGF2 exon 10 was highly methylated (70-100%) at almost all CpG sites in all three tissues examined and in all treatment groups (figure 1). There was a high level of variation in methylation levels between individual samples, even in normal control tissues. Only the adrenal tissues showed a significant difference in mean methylation between AI, SCNT and SCNT-hydrops groups; SCNT-hydrops samples tended to be hypermethylated compared with the AI or SCNT samples. At CpG 5, individual SCNT and SCNT-hydrops adrenal samples were either completely methylated or unmethylated, whereas the AI samples were methylated to between 30-40%. No notable tissue-specific methylation patterns were observed. When averaged over the entire region analyzed, the methylation levels ranged from 65 to 85% for all three tissues (figure 1d). A significant difference was detected in the regional mean methylation levels between the AI and SCNT (P = 0.01) and SCNT-hydrops (P = 0.03) in the adrenal samples, and in the kidney between AI and SCNT (P = 0.04) and between SCNT and SCNT-hydrops (P < 0.001). Three of the SCNT-hydrops samples were almost 100% methylated at every site whilst another was methylated at between 40-50% at almost every site, illustrating the level variation between some individuals in this group.

#### ASCL2

**Figure 2:**
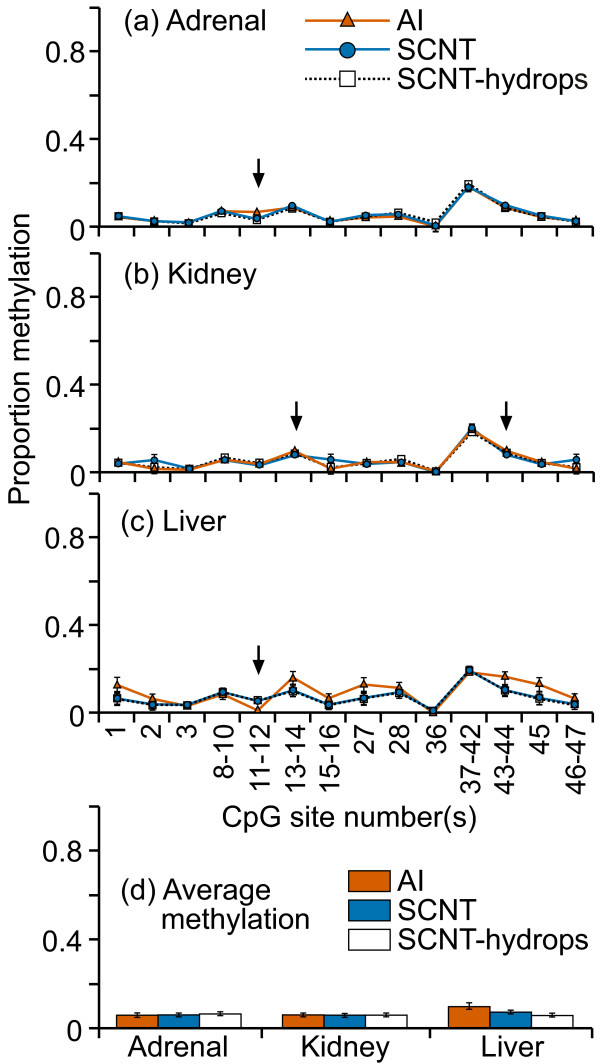
**DNA methylation at the region surrounding the ASCL2 transcription start site**. Fourteen cleavage fragments containing 26 out of a total of 47 CpG sites in this region could be analyzed. The groups of cleavage fragments containing CpG sites 1 and 27 and 45; 2 and 15-16 and 46-47; 13-14 and 43-44 could not be distinguished from each other in the analysis. Therefore, the proportion of methylation in each of these fragment groups is represented as an average value.

Compared with the IGF2 exon 10 region, the DNA methylation levels here were low (less than 25%) in all tissue samples (figure 2). Furthermore, the variation between individuals was very small in all three tissues, resulting in a mean percentage methylation at each CpG site that was remarkably similar between the treatment groups. The fragment containing CpG sites 37-42 was consistently methylated at higher levels (20-25%) compared with other fragments/sites (0-10%) examined in every sample. This difference is likely due to the algorithm used by EpiTYPER to determine methylation levels in fragments containing multiple CpG sites [38]. Despite the apparent similarity in mean methylation levels in the fragments analyzed, significant differences between AI, SCNT and SCNT-hydrops groups were found in certain fragments in all three tissues (figure 2). The mean DNA methylation level across this region was similar in all groups across all tissues (figure 2d).

#### HAND1

**Figure 3:**
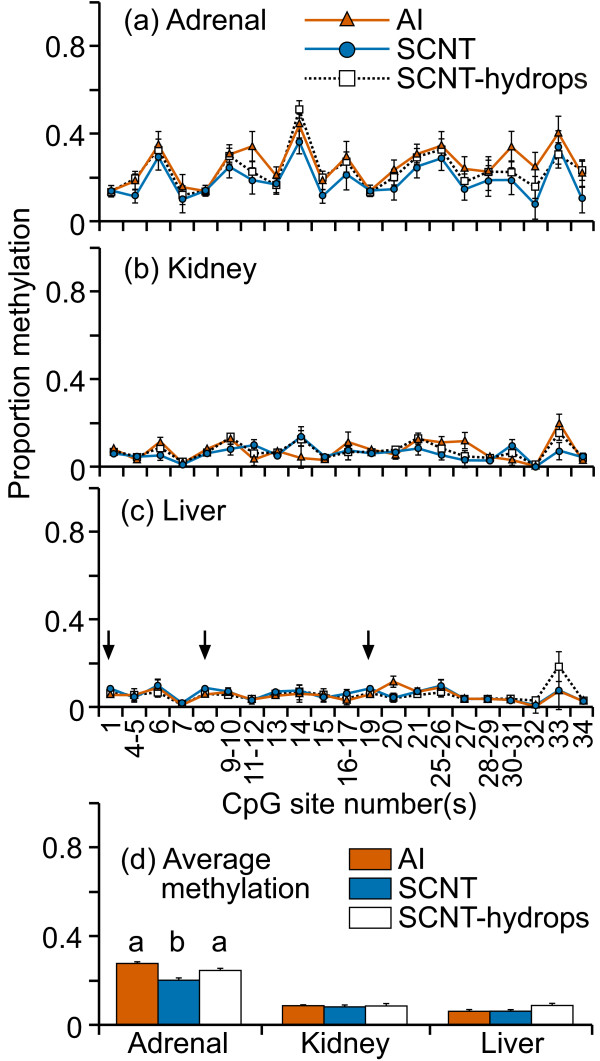
**DNA methylation in the region immediately upstream of HAND1 exon1**. Twenty one cleavage fragments containing 28 out of a total of 34 CpG sites in this region could be analyzed. The groups of cleavage fragments containing CpG sites 1 and 8 and 19; 4-5 and 15; 6 and 25-26; 9-10 and 21; 11-12 and 30-21 could not be distinguished from each other in the analysis. Therefore, the proportion of methylation in each of these fragment groups is represented as an average value.

DNA methylation levels at this CpG island in the promoter region of HAND1 were mostly low (0-20%) in kidney and liver tissues (figure 3). Interestingly, the adrenal tissues from all three groups showed higher methylation levels (10-55%) and greater variability at most CpG sites across this region when compared with either liver or kidney samples. The only statistically significant difference in the mean methylation levels between treatment groups was in the liver (figure 3c); however the difference in methylation levels was very small. Otherwise, DNA methylation levels at individual CpG site/s were not different among the treatment groups. The mean level of DNA methylation across the region was similar between the groups in kidney and liver tissues but a significant difference was observed in adrenal tissue (figure 3d) where SCNT samples were significantly less methylated compared with either AI or SCNT-hydrops samples (P < 0.002). For the adrenal tissues, within the SCNT-hydrops group, there were two individuals cloned from the same donor somatic cell line: one was consistently methylated to between 30-70% at practically every analyzable CpG site whereas the other was methylated to only about 10% for all but three analyzable CpG sites.

#### KCNQ1

**Figure 4:**
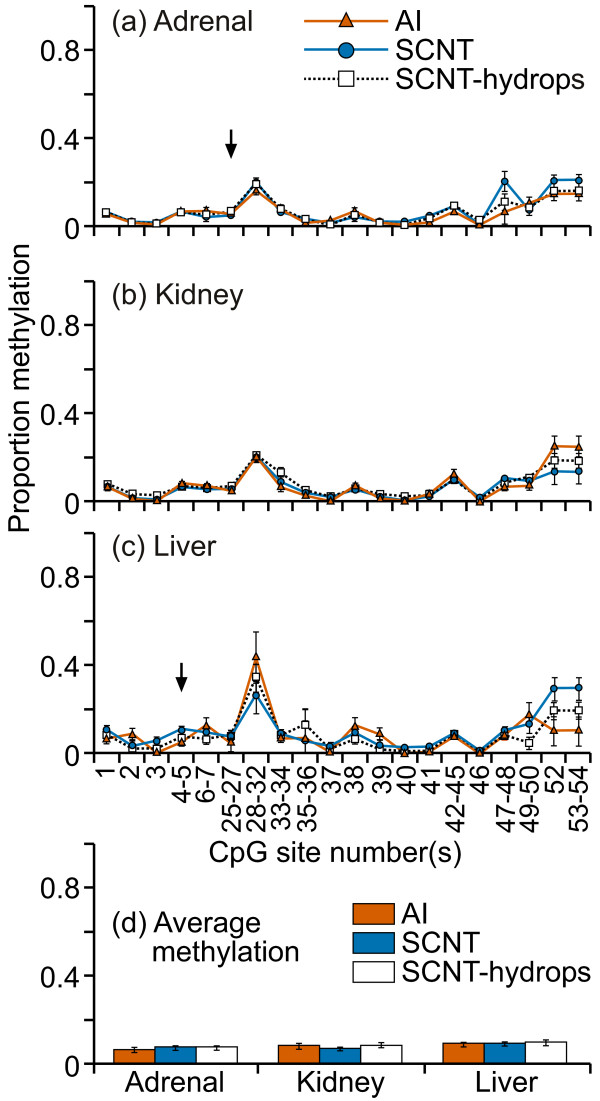
**DNA methylation in the region spanning the putative KCNQ1 transcription start site**. Twenty cleavage fragments containing 36 out of a total of 55 CpG sites in this region could be analyzed. The pairs of cleavage fragments containing CpG sites 2 and 39; 6-7 and 38; 52 and 53-54 could not be distinguished from each other in the analysis. Therefore, the proportion of methylation in each of these fragment groups is represented as an average value.

The percentage of DNA methylation in the KCNQ1 region examined varied from 0-45% across individual CpG sites, with most showing less than 20% methylation (figure 4). Overall, the methylation levels of the samples from all three groups were similar at individual CpG sites in all tissues examined, although significant differences among the groups were detected in some fragments (figure 4a and 4c). No tissue-specific methylation patterns were observed and the homogeneity of the methylation levels in all the samples was reflected in the lack of difference in the mean regional methylation levels between the tissues and groups examined (figure 4d).

#### CDKN1C

**Figure 5:**
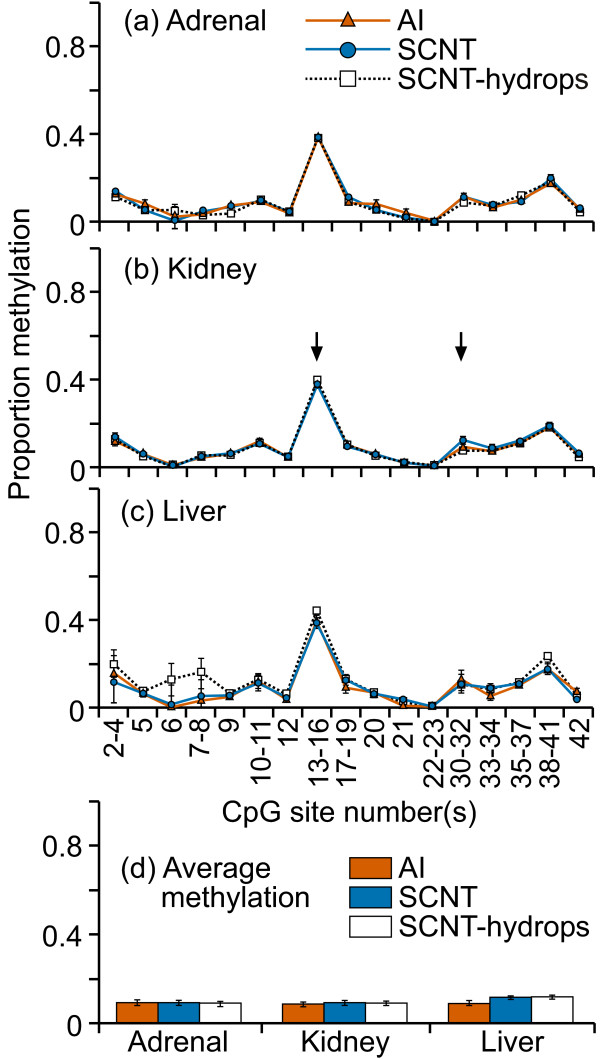
**DNA methylation in the region spanning intron 3 of CDKN1C**. Seventeen cleavage fragments containing 35 out of a total of 42 CpG sites in this region could be analyzed. The cleavage fragments containing CpG sites 5 and 20 could not be distinguished from each other in the analysis. Therefore, the proportion of methylation in these fragments is represented as an average value.

The methylation pattern in this CpG island, which is within the transcribed region of CDKN1C, was very similar across all groups and in all tissues (figure 5). There was little variation at practically every CpG site between individual samples within each treatment group; this lack of variation is reflected in the similarity of the regional mean methylation levels (figure 5d). The mean methylation levels at individual CpG sites or groups of CpG sites were between 0-20% with the exception of one cleavage fragment containing CpG13-16, which was methylated to 40-45% in every group and tissue examined. Because this fragment contains four CpG sites, it was not possible to determine if certain sites were methylated to a greater extent than others. CpG6 and CpG22, 23 were always completely unmethylated in every sample. Due to the limited variation between individual samples in this region, even differences as small as 2-3% showed statistical significance at some sites (figure 5b).

#### SNRPN

**Figure 6:**
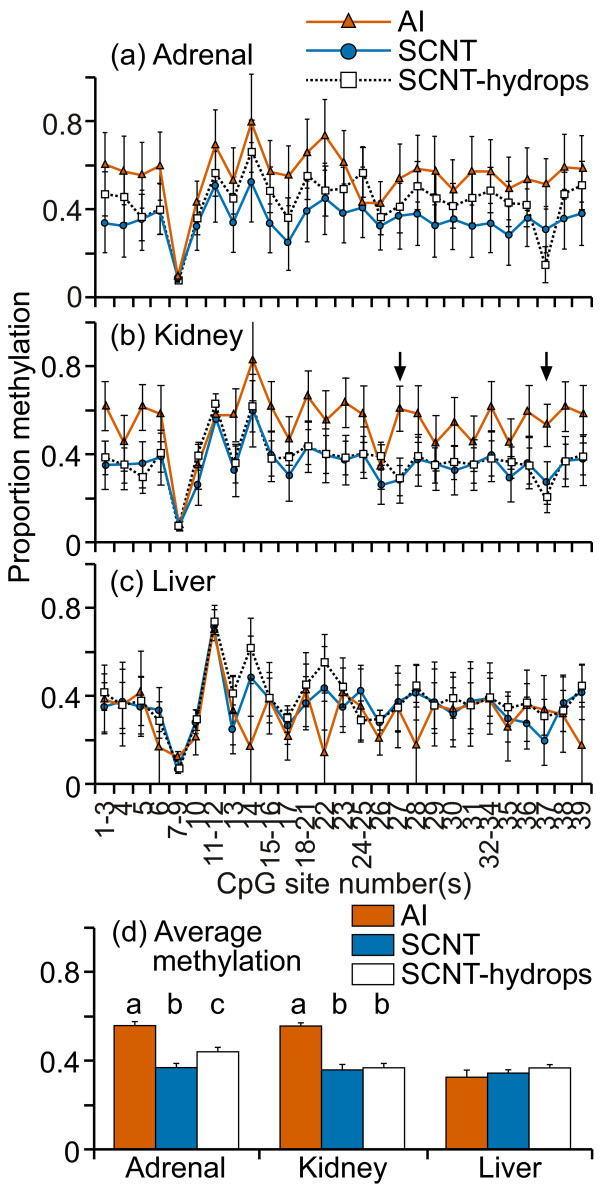
**DNA methylation in the SNRPN exon 1 region**. Twenty-seven cleavage fragments that contained all 39 CpG sites in the region were analyzed. The groups of cleavage fragments containing CpG sites 4, 29 and 31; 10 and 26; 15-16 and 32-34 could not be distinguished from each other in the analysis. Therefore, the proportion of methylation in each of these fragment groups is represented as an average value.

The methylation levels at each CpG site/groups of sites in this region showed a high degree of variation between individuals in all three tissues and in every treatment group (figure 6). Although the majority of the sites showed no significant differences in mean methylation levels between the treatment groups, DNA from AI samples tended to be more methylated than SCNT or SCNT-hydrops, particularly for the adrenal and kidney samples. In these two organs, the methylation levels in the AI samples ranged between 30 - 100% for most CpG sites whereas in the adrenal samples, three each of the SCNT and SCNT-hydrops samples were almost unmethylated (<10%); the others were methylated to a similar extent as the AI samples. Similarly low levels of methylation in individual SCNT and SCNT-hydrops kidney samples were also observed. Only one SCNT and one SCNT-hydrops foetus both showed consistent hypomethylation in all three tissues whilst others that were hypomethylated in one tissue showed almost normal methylation levels in other tissues. The regional mean methylation levels were significantly higher (P < 0.001) in the AI adrenal and kidney but not liver samples when compared with SCNT or SCNT-hydrops (figure 6d). Only two fragments in kidney tissues showed significant difference in mean methylation levels (AI>SCNT, SCNT-hydrops, figure 6b).

A cleavage fragment containing CpG sites 7, 8, and 9 was consistently methylated to ≤10% in every sample analysed. To eliminate the possibility that this was due to unknown SNPs resulting in a C to T conversion and thus loss of CpG sites, a subset of 10 DNA samples were amplified across this region and the amplicons sequenced. All samples contained the expected CGCGCG sequence.

#### KCNQ1OT1

**Figure 7:**
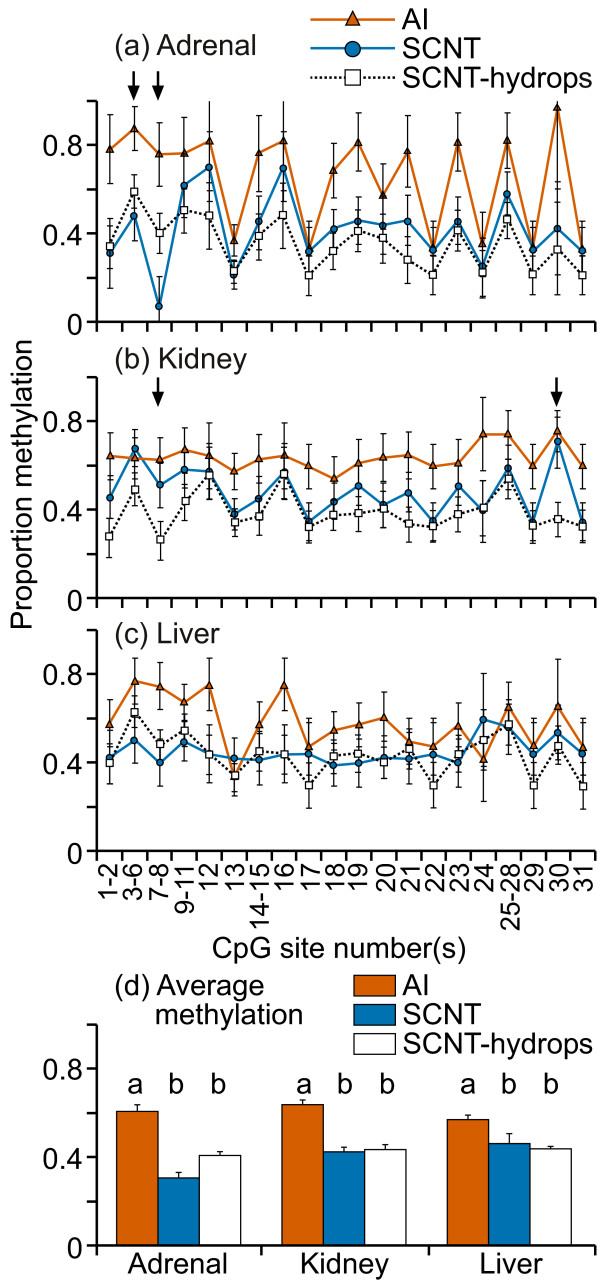
**DNA methylation at the putative KCNQ1OT1 DMR**. Twenty cleavage fragments that contained all 31 CpG sites in the region were analyzed. The groups of cleavage fragments containing CpG sites 12 and 16; 17 and 22 and 29 and 31; 19 and 23, could not be distinguished from each other in the analysis. Therefore, the proportion of methylation in each of these fragment groups is represented as an average value.

The KCNQ1OT1 region examined corresponds to the Kcnq1ot1 DMR in the mouse and human genomes. Similar to the above SNRPN region, the variation in methylation levels between individuals at each CpG site was large for all three tissues (figure 7). DNA from SCNT and SCNT-hydrops foetuses tended to be less methylated in all three organs when compared with AI samples, resulting in significantly lower regional average methylation (figure 7d) in the SCNT and SCNT-hydrops groups (P < 0.001, except the liver, where AI vs. SCNT, P = 0.03). There was no significant difference between the SCNT and SCNT-hydrops groups. Where significant differences were detected between treatment groups at individual CpG sites (figure 7a and 7b), these differences were large compared with the differences seen in CDKN1C and KCNQ1.

#### DIO3

**Figure 8:**
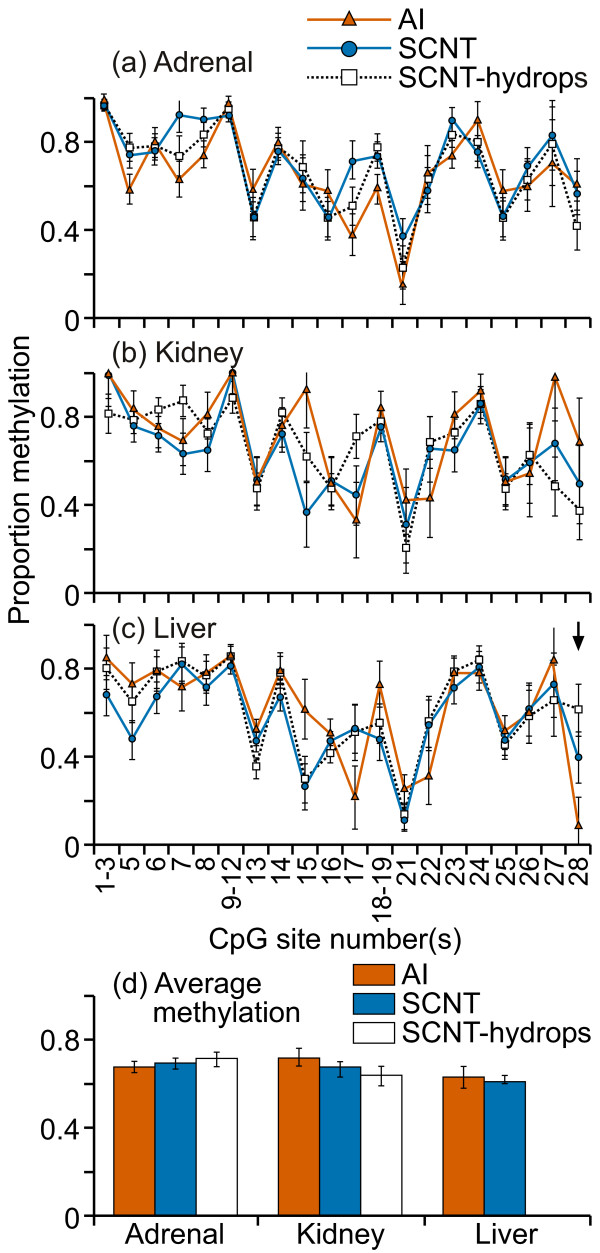
**DNA methylation at the DIO3 polyA signal**. Twenty cleavage fragments containing 26 out of a total of 28 CpG sites in this region could be analyzed.

The DIO3 region examined is a good example of how widely DNA methylation levels at individual CpG sites/fragments can vary within a single CpG island (20-100%). Highly methylated CpG sites appeared to be interspersed between CpG sites that were methylated at low levels (figure 8); this variation was seen in all three tissues and in all treatment groups. Only one CpG site in liver samples showed significant difference among the groups (figure 8c). There were no significant differences among the treatment groups (figure 8d) in regional average DNA methylation.

### Non-imprinted genes

#### GR

**Figure 9:**
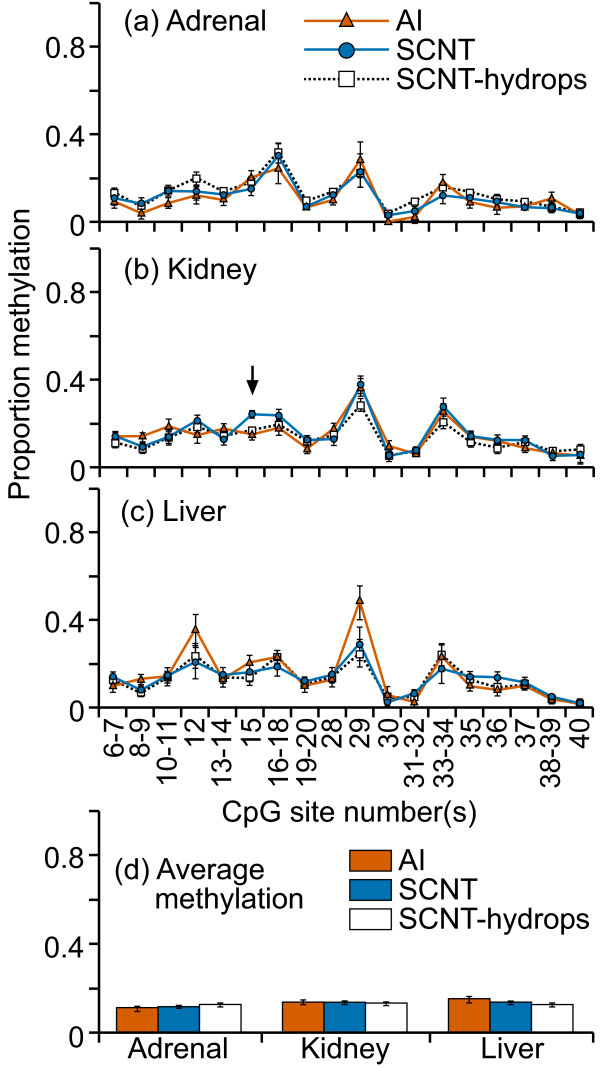
**DNA methylation in the GR promoter**. Eighteen cleavage fragments containing 28 out of a total of 40 CpG sites in this region could be analyzed. The pairs of cleavage fragments containing CpG sites 6-7 and 35; 13-14 and 28; 19-20 and 37 could not be distinguished from each other in the analysis. Therefore, the proportion of methylation in each of these fragment groups is represented as an average value.

Methylation levels in this region were relatively low (0-30% in the kidney and adrenal and 0-50% in the liver) at individual CpG sites (figure 9). Significant difference was found only in the kidney at once CpG site (figure 9b). There were no obvious tissue-specific methylation patterns. The mean regional methylation levels were 10-15% with no difference detected among the treatment groups in any of the tissues (figure 9d).

#### CSF-1

**Figure 10:**
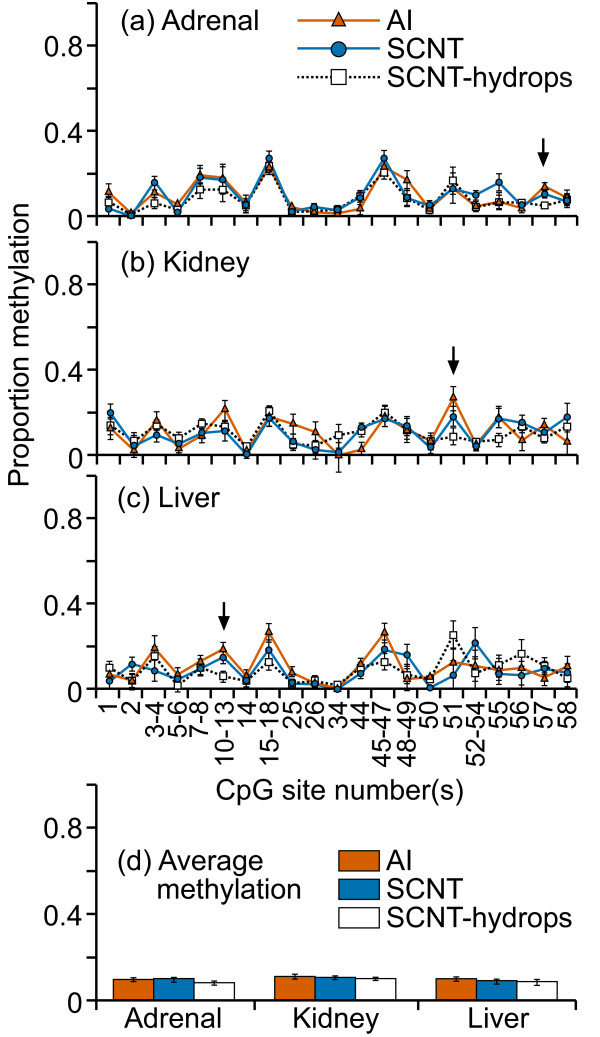
**DNA methylation at the CSF-1 transcription start site**. Twenty-one cleavage fragments containing 35 out of a total of 58 CpG sites in this region could be analyzed. The pair of cleavage fragments containing CpG sites 15-18 and 45-47 could not be distinguished from each other in the analysis. Therefore, the proportion of methylation in each of these fragment groups is represented as an average value.

Mean methylation levels were relatively low across the region analyzed (0-30%) with little variation between individual CpG sites (figure 10) and no tissue-specific methylation patterns were evident. Although mean regional methylation levels were not different among the groups (figure 10d) there were specific CpG sites where significant differences were detected. These specific CpG sites were different for each tissue type (figure 10a, b, c). In all cases, the differences were small.

#### DKK-1

Mean methylation levels were similar to those reported for Day 26 trophoblast tissue [39], with all CpG sites methylated to ~10%, the exception being CpG31, where mean methylation levels were 40-50%. There were no significant differences among the three groups.

#### STAT5a

**Figure 11:**
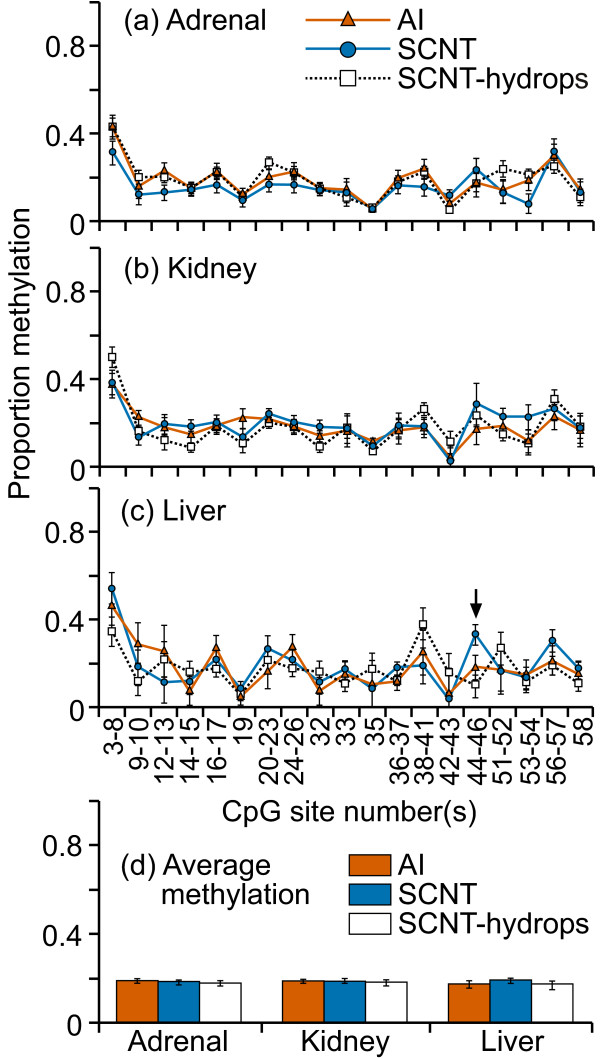
**DNA methylation 10 kb upstream of the STAT5a transcription start site**. Nineteen cleavage fragments containing 43 out of a total of 58 CpG sites in this region could be analyzed. The pairs of cleavage fragments containing CpG sites 14-15 and 32; 16-17 and 24-26; 33 and 58 could not be distinguished from each other in the analysis. Therefore, the proportion of CpGs methylated in each of these fragment pairs is represented as average values.

DNA methylation levels in a CpG island between STAT5a and STAT5b were mostly below 30% in kidney, adrenal and liver samples (figure 11). The only difference between treatment groups was at one CpG site in the liver samples (figure 11c). Individual methylation profiles of liver samples in the SCNT-hydrops group were more variable than those in the AI or SCNT groups with some CpG sites in certain individuals almost completely methylated. No differences were noted between the groups in the mean DNA methylation levels across the region in any of the tissues (figure 11d).

### Repeat regions

#### DNA Satellites I, II and alpha

**Figure 12:**
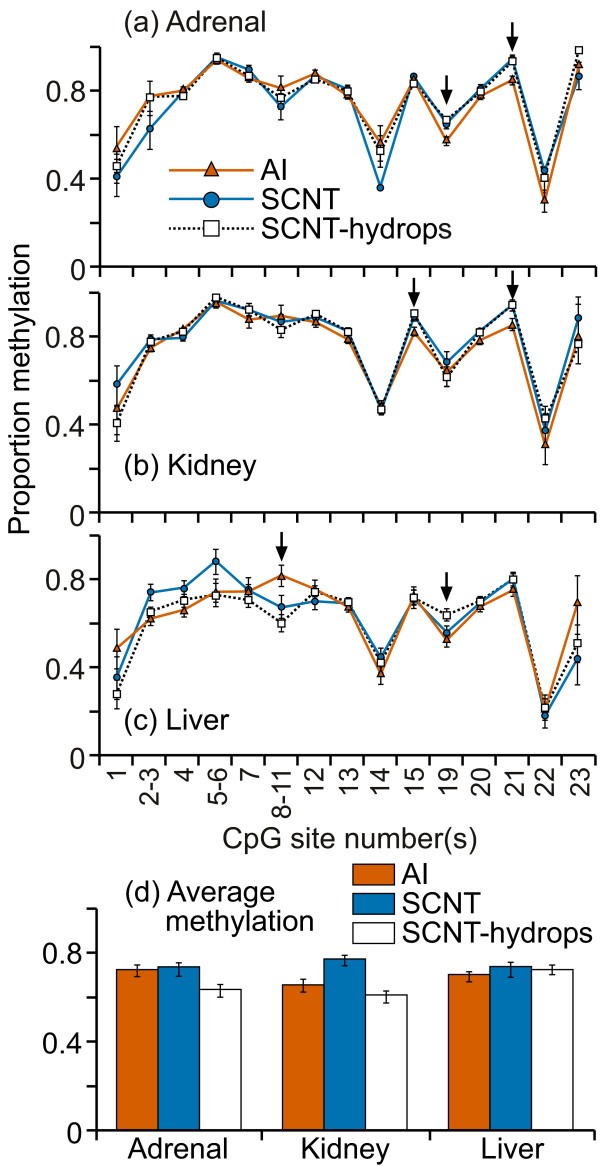
**DNA methylation across satellite I repeat sequence**. Fifteen cleavage fragments containing 20 out of a total of 23 CpG sites in this region could be analyzed. The cleavage fragments containing CpG sites 13 and 20 could not be distinguished from each other in the analysis. Therefore, the proportion of methylation in each of these fragment groups is represented as an average value.

The majority of CpG sites in the Satellite I sequence examined were methylated to > 80% in all adrenal, kidney and liver samples (figure 12). Four of the CpG sites (1, 14, 17, and 22) were consistently methylated to ≤ 50% in all tissue types. CpG sites where the mean methylation levels were significantly different among the groups were observed in all three tissues types (figure 12a, b, c).

**Figure 13:**
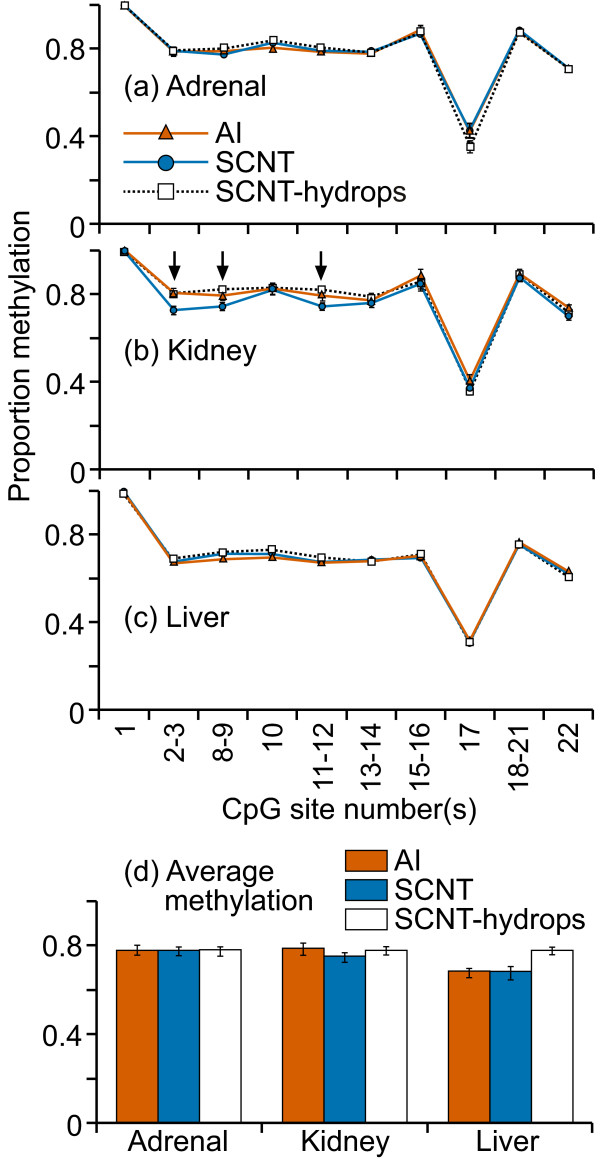
**DNA methylation across satellite II repeat sequence**. Ten cleavage fragments containing 18 out of a total of 22 CpG sites in this region could be analyzed. The cleavage fragments containing CpG sites 8-9 and 11-12 could not be distinguished from each other in the analysis. Therefore, the proportion of methylation in each of these fragment groups is represented as an average value.

The methylation levels and pattern for Satellite II sequences were remarkably similar between individuals, treatment groups and tissue types (figure 13). CpG1 was invariably 100% methylated, CpG17 was methylated to 30-40%. All other CpG sites were 70-80% methylated. Only three cleavage fragments in SCNT kidney samples showed significantly different mean methylation levels compared with the other two groups (figure 13b).

**Figure 14:**
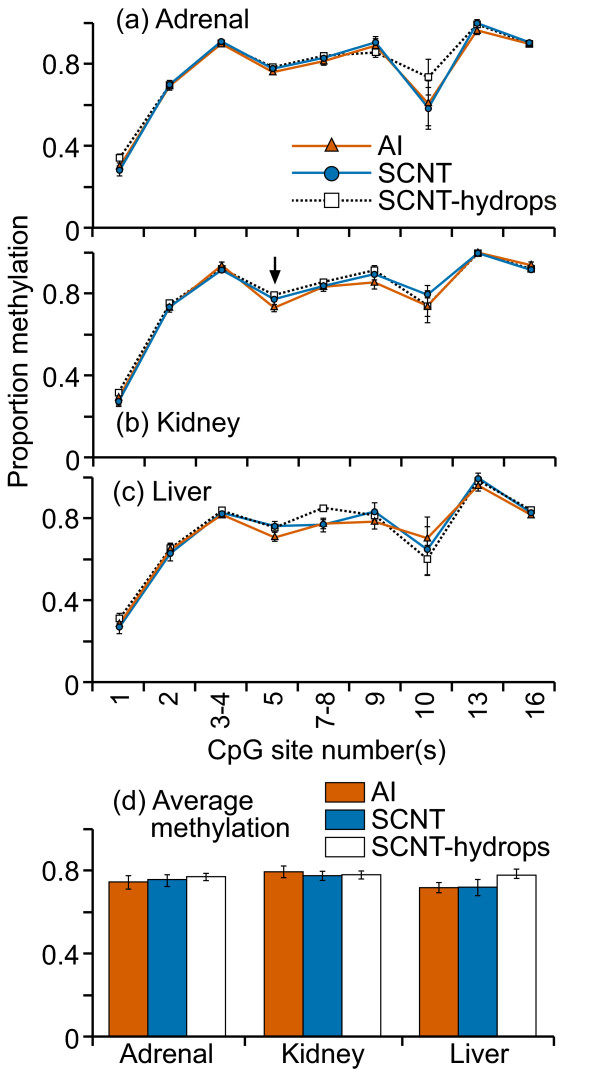
**DNA methylation across satellite alpha repeat sequence**. Nine cleavage fragments containing 11 out of a total of 16 CpG sites in this region could be analyzed. The cleavage fragments containing CpG sites 3-4 and 16 could not be distinguished from each other in the analysis. Therefore, the proportion of methylation in each of these fragment groups is represented as an average value.

The Satellite alpha sequences also showed similar patterns of methylation in all the treatment groups and tissues (figure 14) with the only significant difference being found at one CpG site in the kidney (figure 14b). There were no apparent tissue-specific methylation patterns. Other than CpG10, which showed a moderate level of variation between individuals, the other CpG sites were consistently methylated to the same degree in every sample, tissue and treatment group. CpG 13 was completely methylated in every sample. No significant differences were noted between the three groups in the mean DNA methylation levels across any of the satellite regions in any of the tissues (figures 12d, 13d and 14d).

## Discussion

In this study, we addressed the question of whether nuclear reprogramming has occurred appropriately after SCNT by examining DNA methylation patterns in tissue samples collected from three organs from mid-gestation foetuses. Being able to sub-divide the SCNT group into those that appeared "viable" at slaughter and those that were failing as a result of clinical hydrops (SCNT-hydrops) allowed us to determine if the failing foetuses were methylated differently compared with those that had the potential to develop further. As there are no means to determine, with certainty, the outcome from a foetus that looked "viable" at mid-gestation (SCNT or AI), we can only make the assumption that these SCNT foetuses have the potential, like the AI foetuses, to develop further. Previous studies have indicated that 50-75% of foetuses surviving to mid-gestation will result in a live calf [40-43]. In the majority of regions examined, the DNA from the SCNT or SCNT-hydrops samples were appropriately methylated when compared with control AI samples. Generally, there were no differences in the mean methylation patterns and levels between the SCNT and SCNT-hydrops groups despite evident phenotypic differences. However, close inspection of individual profiles revealed that within the SCNT-hydrops group, there were individuals who showed aberrant hypo- or hypermethylation, particularly in the IGF2 exon 10 DMR, KCNQ1OT1, SNRPN and HAND1 regions. Aberrant methylation in any of those genes was not always accompanied by aberrant methylation in other regions. Furthermore, two individual that are genetically identical could be aberrantly methylated in different ways, demonstrating the stochasticity of the reprogramming defects. At specific CpG sites, significant differences between SCNT and AI samples were detected in some genes. It is not possible to say if these sites represent "hot-spots" for methylation variation or whether these methylation aberrations are the cause of some of the abnormal phenotype seen in SCNT foetuses.

Genes in the BWS locus were of particular interest because of the variable overgrowth phenotypes seen in SCNT foetuses and how some of the phenotypes resemble BWS in humans. The methylation profiles of CpG islands associated with the KCNQ1 and CDKN1C genes were practically identical between the AI control and both groups of SCNT, in liver, kidney and adrenal samples. This suggests that the methylation at these sites is tightly regulated and they were appropriately methylated in these SCNT organs, even in those from failing hydrops pregnancies. Similarly, no differences were observed for the region associated with ASCL2. This tight regulation did not extend to the KCNQ1OT1 region located close to the CDKN1C gene in this imprinted cluster. CpG sites in the KCNQ1OT1 region showed substantial variation between individuals and a tendency for SCNT samples to be less methylated at almost every analyzable CpG site. Taken together, these results suggest that even within the same locus, some CpG islands are methylated appropriately in SCNT tissues while others are less so. As it was not possible to distinguish between the maternal and paternal allele in cattle, we were unable to determine if the aberrant methylation was restricted to one allele or if both were aberrantly methylated.

Similarly, at the region corresponding to the SNRPN imprint control region in humans, the methylation levels were also highly variable between individuals in all groups. Mean methylation at the majority of CpG sites in both SCNT groups tended to be lower when compared with the AI group, although not as dramatically hypomethylated as in early extraembryonic tissues, (Couldrey and Lee, unpublished data and that of others [25]). Although there was a tendency for this region to be incorrectly methylated following SCNT, not all CpG sites in this region were affected in the same way. An example of this is the cleavage fragment containing CpG7-9 which was consistently unmethylated for every sample in each of the three tissues in all treatment groups. It is possible that these CpG sites are invariably protected from epigenetic modification.

Small but statistically significant differences in methylation levels between SCNT and the control group were detected at specific CpG sites in HAND1, ASCL2 and the KCNQ1 promoter regions in various tissues. These differences were very small and the overall methylation status of the entire region examined was not altered so their biological significance is uncertain. The occurrence of these very small differences is no higher than would be expected purely by chance at the 5% level. Only the HAND1 region showed tissue specific methylation differences as the adrenal tissues have a noticeably different pattern compared with the other two tissues. In these regions, the similarity in methylation pattern between SCNT and normal tissues suggests that this region has undergone appropriate reprogramming and that tissue-specific methylation patterns were successfully established in this organ. Where there were no tissue-specific methylation differences, the absence of difference in methylation between AI and SCNT samples is either an indication of successful reprogramming or that these regions normally escape global demethylation [23,29,44] in the pre-implantation embryo and therefore did not require extensive reprogramming after SCNT.

For imprinted genes, the imprints that mark the parental origin of each allele are normally established during gametogenesis, leading to differential methylation of the male and female gametic DNA in DMRs. The methylation levels in DMRs have previously been reported to be ~50%. This level of methylation was believed to be due to the almost complete methylation of one parental allele versus the non-methylation of the other allele. We found that for the IGF2 exon 10 DMR, the majority of the CpG sites were methylated to between 70 to nearly 100% in the three foetal tissues examined, even in the controls. Assuming that the paternal allele was completely methylated, this suggests that the maternal allele was progressively methylated during development. One possible explanation for this is that after additional epigenetic marks that dictate allele-specific expression are established during early embryogenesis, there is no longer the requirement to maintain the differential marking at the DMRs so most of the CpG sites in somatic cells then become progressively methylated. Alternatively, it may be that only a few CpG sites in the region are required to be differentially methylated to distinguish the parental alleles so the non-essential sites become methylated through methylation spreading.

Comparison of the methylation profiles in non-imprinted genes and the repetitive DNA satellite regions showed that in general, these regions appeared to be appropriately methylated in the SCNT tissue samples examined. Although satellite sequences are non-coding and are thought to be kept highly methylated in the genome, not every CpG site was methylated to the same extent across the region. Despite significant differences in methylation levels at specific CpG sites between treatment groups, these differences are very small and the significance in unclear.

An intriguing observation from this study is that in some genes, there is a surprisingly large variation in the methylation levels between individuals at practically every CpG site in the region examined, even between individuals in the normal control group. This suggests that there is tolerance for a range of DNA methylation levels in some genomic regions; whether this translates to phenotypic variability is unknown. This variability could be in part, explained by the inherent lower fidelity (compared with DNA replication) of the DNA maintenance methylation mechanism, which is estimated to be about 95% for methylation of the newly unmethylated strand [45]. However, this does not explain why the methylation of certain regions is so tightly regulated. This variability is not due to heterogeneous tissue sampling as the analyses for multiple genes were carried out on the same bisulfite-treated DNA sample for each individual. In these same samples, other genomic regions showed very tight invariant methylation at almost all CpG sites in every individual. In general, consistent with previous findings [45] CpG islands upstream of transcriptional start sites or near promoters were less methylated (5-20%) than the two putative DMRs and satellite sequences, which tended to be methylated to 40-100%.

Previous studies assessing the reprogramming of the donor nucleus after SCNT have used antibodies raised against methylcytosine residues to compare global methylation in the nuclei of SCNT and normal embryos [27]. This technique only allows visualization of highly methylated regions which are likely to be repeat sequences that do not code for functional genes. Subtle differences in regions present at two copies per genome will be masked by the overall methylation of highly repetitive elements. Other techniques which average DNA methylation levels over the genomic region analyzed have led to the misconception that DNA methylation levels are similar at each CpG site across the entire CpG island. In contrast, the MassARRAY technology is able, in many instances, to calculate DNA methylation levels at individual CpG sites reproducibly down to 5% for each informative CpG unit [38]. However, because the MassARRAY method depends on sequence-specific cleavage of derived RNA products, this technology is unable to analyze the methylation at every CpG site when suitable cleavage sites are unavailable such as in high density CpG regions. Bisulfite sequencing will be a useful adjunct when these regions warrant further examination. The success of both techniques however, is dependent on being able to design primers that flank ~500 bp of CpG-rich sequences but the primers themselves must bind to regions which do not contain CpG sites. For some CpG islands that are large (up to ~8 kb), this is not always possible and other methods must be devised to study such regions.

The ability to analyze large numbers of samples and genes and quantify the level of methylation at specific CpG sites allows a more accurate assessment of methylation profiles in the populations of interest. The technology has enabled us to appreciate detail previously unrealized: a) some CpG sites are always protected from methylation whilst others tend to be highly methylated; b) methylation at some CpG site(s) within a region show high variability between individuals whilst others are invariably methylated in every individual; c) some regions, such as the KCNQ1OT1 site in adrenal tissues and DIO3 in all tissues show apparent periodicity in the methylation profile, with highly-methylated sites interspersed with lowly-methylated sites; d) tissue-specific methylation patterns were uncommon. It remains for the biological significance of these observations to be determined.

The detail revealed by this method of DNA methylation analysis calls into question whether the practice of reporting the average methylation level across all CpG sites within a region and comparing this value between experimental samples is meaningful. This could potentially mask methylation differences between experimental groups at CpG sites that may be important for regulating chromatin structure and hence, gene expression. CpG sites that are invariably protected from methylation, or those which are always methylated are not evident when averaging methylation levels across a region. Subtle tissue-specific differences may also be masked. Furthermore, the biological significance of averaged methylation levels in a region is unclear.

## Conclusions

We have used the MassARRAY technology to look at multiple regions in the genome and found that for SCNT foetuses that survived to mid-gestation, albeit with phenotypic abnormalities in some cases, the methylation patterns were very similar to those of naturally conceived foetuses, at least for the three organs examined. This suggests that in those foetuses, the majority of these sites in the genome have been appropriately "reprogrammed". However, there were two regions located in imprinted gene clusters (BWS and PWS loci) where SCNT samples tended to be hypomethylated. This implies the importance of these two region in regulating normal foetal development and growth. Similar observations of aberrant methylation in the BWS locus in children arising from human IVF is further evidence of the susceptibility of this region to external influence. We cannot yet with confidence predict the developmental outcome of a SCNT foetus from its epigenetic state at any stage; it is just a snap-shot of the dynamic nature of the epigenetic status of the genome. We are far from understanding how DNA methylation patterns relate to phenotypic outcomes in entire organisms.

## Methods

### Production and collection of foetal tissues

All manipulations of animals involved in the present study were conducted in accordance with the regulations of the New Zealand Animal Welfare Act of 1999. SCNT embryos were produced essentially as previously described [40]. An adult skin fibroblast cell line (AESF-1) from a high genetic merit Friesian bull was used as nuclear donor. After in vitro culture for 7 days, the embryos were transferred to synchronized recipients and pregnancy establishment determined at Day 35 of gestation by trans-rectal ultrasound scanning. Pregnancies were monitored monthly by ultrasound scanning until Day 120. From then, the animals were monitored closely by rectal palpation for the development of hydrallantois. SCNT pregnancies that were diagnosed with hydrops were terminated by slaughter at the abattoir. Viable SCNT pregnancies from the same cohort but without hydrops were also terminated at around the same gestation as those with hydrops. Additional samples from SCNT-hydrops pregnancies at similar stages of gestation generated from several other donor cell lines were included in the study. Thus, the group SCNT-hydrops consisted of SCNT foetuses derived from the AESF-1 line as well as from five other cell lines of both sexes and of different breeds. This allowed us to investigate whether the DNA methylation status is similar or different in SCNT-hydrops foetuses with different genetic backgrounds. Control pregnancies were generated by artificial insemination (AI) with frozen semen from the bull which provided the AESF-1 donor cells and foetal tissues were collected at the equivalent stage of gestation as the SCNT foetuses. The uteri and its contents were recovered after slaughter of the recipient dams. Gross foetal and placental morphology was recorded and foetal and placental tissue samples collected and snap-frozen in liquid nitrogen. For this study, three organs, the kidney, liver and adrenal glands, which commonly show growth disregulation in SCNT foetuses, were used (AI, n = 5; SCNT, n = 6 and SCNT-hydrops, n = 4 from the AESF-1 line and n = 6 from other cell lines).

### Identification of CpG islands for analysis

Promoter regions (up to 10 kb upstream of the putative transcription start site) and transcribed regions of selected genes were analysed for the presence of CpG islands (observed/expected CpG dinucleotide ratio of > 0.60, C+G content > 50%, length > 200 bp) using Emboss on EBI website.

Primers were designed (table 1), using MethPrimer, to flank and amplify CpG island sequences in genes of interest, as described [46]. Primer sequences contained at least four Cs that were not in CpG pairs and no CpG sites. The regions of interest chosen include: a) a region spanning the putative transcription start site of ASCL2, a gene associated with placental development; b) a region in exon 10 of IGF2 (GenBank accession no. X53553) that is differentially methylated [47]; c) a region spanning the putative transcription start site of KCNQ1; d) 500 bp of the CpG island corresponding to the human KCNQ1OT1 DMR; e) 1-0.5 kb upstream of exon 1 of HAND1, coding for a transcription factor associated with trophoblast differentiation, cardiogenesis and the development of neural crest derivatives; f) part of exons 3 and 4 and intron 3 of CDKN1C; g) a region beginning upstream of exon 1, covering exon 1 and part of intron 1 of SNRPN (GenBank accession no. AY743660); h) a region at the transcriptional start site for the DIO3 antisense transcript, covering the polyA signal of DIO3 gene, which codes for iodothyronine deiodinase type 3; i) a CpG island (covered by two non-overlapping amplicons) at the transcriptional start site of DKK-1, a potent inhibitor of the WNT signalling pathway which is highly expressed in mesenchymal lineages and may mediate the inductive interactions between the mesenchyme and the epithelium; j) a region 10 kb upstream of the transcriptional start site of STAT5a, a signaling protein for many cytokines and growth factors; k) a region in the bovine GR gene equivalent to the rat exon 1_7 _promoter region, previously shown to be epigenetically modified in rat pups by maternal behaviour towards them [48-50]; l) a region near the transcriptional start site of CSF-1, a cytokine implicated in the development of certain haematopoietic cell lineages; m) a region spanning a CpG island in satellite sequence I (GenBank accession no. J00032); n) a region spanning a CpG island in satellite sequence II (GenBank accession no. X03116); and o) a region spanning a CpG island in satellite sequence alpha (GenBank accession no. AJ293510). The regions chosen for KCNQ1OT1, GR, IGF2 exon 10 DMR, and SNRPN were those where DNA methylation had previously been analyzed in the human, mouse, rat or cow. For ASCL2, KCNQ1, DIO3, DKK1 and CSF1, where there have been no previous data, we chose to analyze DNA methylation in CpG islands near the transcription start sites. Due to the high C+G content and density of CpG sites in the CDKN1C gene, it was not possible to design primers that could be used with bisulfite-converted DNA that would span < 500 bp in the promoter region. The intragenic region analyzed in CDKN1C was as close to the promoter as possible.

**Table 1:** PCR primers used for Sequenom analysis

Name	Sequence	Chromosomal location	Product size (bp)
SNRPN_L	AGGAAGAGAGTTGGGAGGTATTATTTTGGGTTGAA	ChrU:24360-23919	437
		
SNRPN_R	CAGTAATACGACTCACTATAGGGAGAAGGCTAACCCCAAACCTCCAAAAATTATC		

IGF2_L	AGGAAGAGAGGGGTATTTGGGGTAGTTTAGG	Chr29:3633319-3633749	521
		
IGF2_R	CAGTAATACGACTCACTATAGGGAGAAGGCTATTCTAATCCCCTCAACCAAATAAA		

CDKN1C_L	AGGAAGAGAGGTAGTGGTATATTTAGTTGGAAGTTGTAGT	Chr29:2954381-2953897	485
		
CDKN1C_R	CAGTAATACGACTCACTATAGGGAGAAGGCTTAGTTAGGTTAGAGTTAGTT		

HAND1_L	AGGAAGAGAGGAGAAAGGTTTTTGGGGATAAAATT	Chr7:1101938-1101390	549
		
HAND1_R	CAGTAATACGACTCACTATAGGGAGAAGGCTCAAACCCTACAACTAACAAAACATCC		

ASCL2_L	AGGAAGAGAGGTATTAGGGGGAGTTTTGGTAG	Chr29:3526331-3526685	354
		
ASCL2_R	CAGTAATACGACTCACTATAGGGAGAAGGCTCTAAAACCCCAAATTCACCAACTTC		

KCNQ1_L	AGGAAGAGAGGGGTTTGGTTAAGAAGTGTTTTTTTT	Chr29:3365714-3365215	500
		
KCNQ1_R	CAGTAATACGACTCACTATAGGGAGAAGGCTAATCAAACCCACAAAACCCTAAACTT		

Dio3_L	AGGAAGAGAGTTTGTATTTGTTTGGTTTGTTTTAA	Chr21:1453786-1454238	453
		
Dio3_R	CAGTAATACGACTCACTATAGGGAGAAGGCTCAACTCTTCATCAACAATAAAACTC		

KCNQ1OT1_L	AGGAAGAGAGTAGTTGATTGGATAGTTTGTAGGGG	Chr29:3133697-3133352	346
		
KCNQ1OT1_R	CAGTAATACGACTCACTATAGGGAGAAGGCTCCACAAATATTCCTCAAAATCACTC		

STAT5_L	AGGAAGAGAGTTTGTTAGAGGTAGTTGATTTTTGAGGA	Chr19:872990-873467	478
		
STAT5_R	CAGTAATACGACTCACTATAGGGAGAAGGCTAAAAAAACAAAACACTCCCTCTCTC		

GR_R	CAGTAATACGACTCACTATAGGGAGAAGGCTAATTTTCTCTATAATTTCTCTTCTTACC	Chr7: 2747426-2747103	324
		
GR_L	AGGAAGAGAGTTTTTTTGAAGTTTTTTTAGAGGG		

DKK_001_L	AGGAAGAGAGTTTTTTTTGAGTTTTTTTGAGATGA	Chr26:336954-336488	467
		
DKK_001_R	CAGTAATACGACTCACTATAGGGAGAAGGCTCACTTAAACACCCAATACCACACT		

DKK_002_L	AGGAAGAGAGGTGTGGTATTGGGTGTTTAAGTGT	Chr26: 336510-336157	534
		
DKK_002_R	CAGTAATACGACTCACTATAGGGAGAAGGCTCCTAAAATCCTTTCTAAAAATCCTC		

CSF-1_L	AGGAAGAGAGGTAGTTTTTGGAGTAGTTGTAGGGT	Chr3: 629601-630161	561
		
CSF-1_R	CAGTAATACGACTCACTATAGGGAGAAGGCTCAAAATAATTTCCCATAAACCACATAC		

Satellite I_L	AGGAAGAGAGTGTAGATTGGGGATAGGAGAGTTAG	N/A	345
		
Satellite I_R	CAGTAATACGACTCACTATAGGGAGAAGGCTCCTACTTTATCTAAAAAAAATTACCTTCC		

Satellite II_L	AGGAAGAGAGTTTGGTTTTAGGTTGGGAGTTTAAAG	N/A	278
		
Satellite II_R	CAGTAATACGACTCACTATAGGGAGAAGGCTAAAACAACAATCAAACACCACTCAC		

Satellite alpha_L	AGGAAGAGAGTTTTTTTTGATTTGGATAGGAGGG	N/A	279
		
Satellite alpha_R	CAGTAATACGACTCACTATAGGGAGAAGGCTACTATATTTAAAACCAAAAATTTTTCC		

### DNA extraction

Tissues were ground up in liquid nitrogen to a powder to ensure homogeneity for DNA sampling. Between 20 and 100 mg of tissue was then used for DNA extraction using either phenol/chloroform [51] or a DNeasy kit following the manufacturer's protocol (Qiagen, Austin, TX). DNA concentration and purity was measured using the Nanodrop spectrophotometer (Thermo Scientific, DE, USA)

### Analysis of DNA methylation

DNA samples were analyzed using the methods described [38,52,53]. Briefly, 1 μg DNA was bisulfite treated using the EZ-96 DNA Methylation gold kit (Zymo, CA, USA) to produce methylation-dependent sequence variations of C to T and regions of interest were amplified using T7 tagged PCR primers. PCR conditions were: 200 nM of forward and reverse primers, 200 μM of each dNTP, 1× Qiagen HotStar buffer, 0.2 U Qiagen HotStar Taq polymerase and 2 μl bisulfite converted DNA per reaction in a total volume of 10 μl. PCR cycling conditions were: 94°C 15 min followed by 45 cycles of 94°C, 20 sec; 56°C, 30 sec; 72°C, 1 min with a final extension at 72°C for 3 min. PCR products were analyzed by agarose gel electrophoresis to confirm successful amplification. *In vitro *amplification and transcription was performed on the reverse strand using 2 μl of PCR product using T7 DNA and RNA polymerases and a simultaneous U specific cleavage by RNAse A. Approximately 20 nl of each sample was spotted onto Sequenom MassARRAY chips and subject to mass spectrometry. The efficiency of bisulfite conversion was determined by assessing the quality of the raw data. Incomplete bisulfite conversion generates mass peaks at a mass/charge ratio of 16, 32, 48, etc. greater than the expected peaks, in addition to the expected peaks. Such data were infrequent and excluded from the analyses.

### Statistical analysis

Spectra were analyzed using proprietary peak picking and signal-to-noise ratio calculations. The relative methylation of the CpG sites was then calculated (EpiTYPER, Sequenom, CA, USA) by dividing the peak intensity (area under the peak) of the fragment representing the original methylated DNA, by the sum of the intensities of the peaks representing both methylated and non-methylated DNA. Mean DNA methylation levels for each fragment were compared using the least significant differences calculated from the analysis of variance across the three treatment groups in each tissue examined. The mean methylation level across the region contained in the amplicon was also calculated for each gene and compared pair-wise between treatment groups for each tissue using the t-test. Results are presented as mean +/- standard error of the mean (S.E.M).

## Authors' contributions

CC designed and carried out the DNA methylation studies and participated in the data analysis and drafting of the manuscript. RSFL conceived the study and generated the foetuses for the tissue collection, participated in data analysis and drafting of the manuscript. All authors read and approved the final manuscript.

## Note

DNA methylation levels at CpG sites in the amplicons

Each CpG site or groups of sites which could be analyzed by Sequenom MassARRAY are arranged in the order that they appear in the DNA sequence, 5' to 3' on the x-axis. Where there are more than one CpG sites in a fragment, the numbered CpG sites are grouped together in one position on the x-axis and the proportion of methylation refers to the most methylated site (Sequenom EpiTYPER 1 software). The y-axis represents the proportion of methylation at specific CpG sites in the region analyzed. The error bars represent the SEM and arrows indicate CpG sites where there is significant difference (P < 0.05) among the treatment groups. a) adrenal, b) kidney and c) liver tissues, d) average methylation in the genomic region represented by the amplicon; columns with different labels are significantly different from each other (P < 0.05). AI: AI controls; SCNT: SCNT samples from apparently viable foetuses; SCNT-hydrops: SCNT samples from foetuses terminated because of hydrops.
